# Assessing Fluid Intolerance with Doppler Ultrasonography: A Physiological Framework

**DOI:** 10.3390/medsci10010012

**Published:** 2022-02-09

**Authors:** Jon-Emile S. Kenny

**Affiliations:** 1 Health Sciences North Research Institute, 56 Walford Rd., Sudbury, ON P3E 2H2, Canada; jon-emile@heart-lung.org; 2 Flosonics Medical, 325 Front Street, 4th Floor, Toronto, ON M5V 2Y1, Canada

**Keywords:** point-of-care ultrasound, Doppler ultrasound, fluid responsiveness, fluid tolerance, Starling curve, hemodynamics, physiology, review

## Abstract

Ultrasonography is becoming the favored hemodynamic monitoring utensil of emergentologists, anesthesiologists and intensivists. While the roles of ultrasound grow and evolve, many clinical applications of ultrasound stem from qualitative, image-based protocols, especially for diagnosing and managing circulatory failure. Often, these algorithms imply or suggest treatment. For example, intravenous fluids are opted for or against based upon ultrasonographic signs of preload and estimation of the left ventricular ejection fraction. Though appealing, image-based algorithms skirt some foundational tenets of cardiac physiology; namely, (1) the relationship between cardiac filling and stroke volume varies considerably in the critically ill, (2) the correlation between cardiac filling and total vascular volume is poor and (3) the ejection fraction is not purely an appraisal of cardiac function but rather a measure of coupling between the ventricle and the arterial load. Therefore, management decisions could be enhanced by quantitative approaches, enabled by Doppler ultrasonography. Both fluid ‘responsiveness’ and ‘tolerance’ are evaluated by Doppler ultrasound, but the physiological relationship between these constructs is nebulous. Accordingly, it is argued that the link between them is founded upon the Frank–Starling–Sarnoff relationship and that this framework helps direct future ultrasound protocols, explains seemingly discordant findings and steers new routes of enquiry.

## 1. Introduction

All happy circulations are alike; each unhappy circulation is unhappy in its own way. That is to say, the circulation in its normal, untroubled state maintains constant blood flow and pressure to ensure adequate tissue oxygen delivery. On the other hand, hemodynamic discontent arises from a myriad of insults; when extreme, the circulation cannot sufficiently maintain tissue oxygen supply, which heralds the presence of clinical shock [1]. The most frequent cause of shock is sepsis, when infection triggers life-threatening organ dysfunction [2,3]. Though highly mortal [4], early recognition and therapy is associated with reduced risk of death in sepsis and septic shock [5]. Despite advancements and regularly updated, international treatment guidelines [6], the optimal therapeutic approach is controversial [7,8]—especially with regards to intravenous (IV) fluids [9,10].

Early IV fluid resuscitation has been emphasized in all iterations of the Surviving Sepsis Campaign guidelines [6]. Accordingly, when confronted with signs of tissue hypoperfusion, such as confusion, low urine output, tachycardia, hypotension, etc., clinicians often reach first for IV fluids. Fundamentally, the rationale is to engage the Frank–Starling mechanism of the heart—augmenting volume to the heart improves volume from the heart [11]. In turn, increased arterial blood volume raises mean arterial blood pressure (as a function of vascular impedance); increased arterial pressure then acts as an energy source for perfusing tissue beds, depending on their local metabolic needs [12,13].

However, overzealous IV fluid resuscitation is associated with poor patient outcome and attempts are being made to clarify patient subsets at particular risk [14,15,16,17,18]. Initially, the invasive monitoring of cardiac filling pressures guided IV fluid administration, but the central venous pressure (CVP) and pulmonary artery occlusion pressure (Ppao) neither definitely describe a patient’s volume status, nor how stroke volume (SV) will respond to additional preload [19]. Countering the shortcomings of invasive monitoring in the intensive care unit (ICU), critical care echocardiography (CCE) has become the 21st century monitoring paradigm [20].

The goal, herein, is to concisely review the concepts of ‘basic’ versus ‘advanced’ CCE, as well as fluid ‘responsiveness’, ‘tolerance’ and Doppler ultrasound. Thereafter, the Frank–Starling–Sarnoff relationship is used to link these concepts, explain common physiological misconceptions, encourage hemodynamic investigation based upon first principles and introduce new technology that may enable novel lines of research. To begin this cursory review, MEDLINE and Google Scholar were searched, using the terms “point-of-care ultrasound”, or “critical care echocardiography”, or “Doppler ultrasonography”, or “shock”, from 2015 until present day. Case reports, pediatric studies and lung-focused publications were excluded, leaving 35 publications for review—these were considered along with their bibliographies.

## 2. Basic Critical Care Echocardiography and Its Caveats

**Figure 1:**
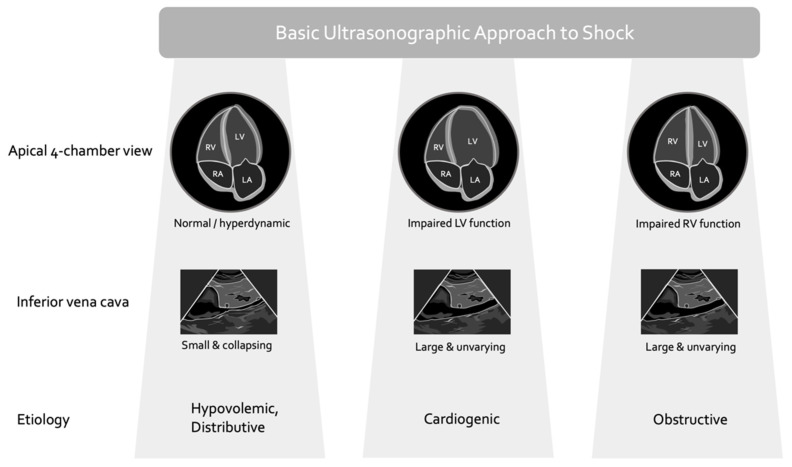
Basic ultrasonographic approach to shock. RA and LA are right and left atria; RV and LV are right and left ventricles. These are simplifications, obstructive shock may be due to pericardial effusion or tension pneumothorax in addition to pulmonary emboli or severe acute respiratory distress syndrome (ARDS). Additionally, shock is often multifactorial; e.g., a patient with septic shock from pneumonia complicated by severe ARDS may have all 4 etiologies occurring in concert.

Pioneering work in CCE began in the late 20th century [21], forming the foundation for many of the contemporary protocols that are often designated by catchy mnemonics; for example, RUSH (rapid ultrasound in shock) [22], FAST (fast assessment in shock and trauma) [23], ACES (abdominal and cardiac ultrasound in shock) [24], EASy (echocardiography assessment using subcostal view only) [25] are described. Virtually all of these approaches are employed to help diagnose the underlying mechanism of shock and, by extension, direct therapy. As described by Repessé and colleagues [20], most of these protocols classify as ‘basic CCE’ because they are usually morphological, rather than functional, assessments. In other words, they rely largely upon image-based evaluation, with less emphasis on quantifiable ultrasound measures (Figure 1).

Each of the aforementioned protocols typically begin with a qualitative appraisal of the inferior vena cava (IVC) and gross approximation of the left ventricular ejection fraction (LVEF) as important steps to narrow shock etiology (Figure 1). Certainly, structured, qualitative, image-based CCE is useful in the differential diagnosis of circulatory failure [26,27], but extending morphological findings to specific therapy, such as IV fluids, should give the clinician pause [28]. Basic CCE generally ignores the following fundamental concepts in cardiac physiology:

(1.) the relationship between cardiac filling and stroke volume (i.e., the Frank–Starling mechanism) varies significantly, especially in the critically ill;
(2.) the relationship between cardiac filling and total vascular volume (i.e., volume status) is poor;
(3.) the LVEF is not purely a gauge of cardiac function but rather a measure of energetic coupling between the ventricle and the arterial load.

Considering the aforementioned, while it is tempting to infer that inspiratory IVC collapse and/or a normal ejection fraction necessarily indicate IV fluids in circulatory failure, these assumptions can be wrong. Inspiratory IVC collapse has multiple co-varying determinants, including changing pleural, abdominal and central venous pressures [29,30,31,32]. Assuming that the pleural and abdominal pressures change consistently (within a patient and between patients) then the primary hemodynamic determinant of IVC collapse is the right atrial pressure or CVP [33]. As elaborated below, the CVP does not reliably speak to how the heart will respond to additional IV fluid [34]. Furthermore, the CVP cannot define total vascular volume (i.e., volume status) [35]. Thus, IVC variation as a surrogate for CVP is not a definitive guidepost for IV fluid [36].

With respect to the LVEF, because it conveys the interaction between ventricular function and arterial load, in a patient with depressed inotropy (e.g., septic cardiomyopathy) and proportionally diminished arterial load (e.g., septic vasodilation), the observed LVEF can be remarkably preserved [37,38,39]. Such a patient may not tolerate additional IV fluids, despite normal-appearing ventricular behavior. Further, LVEF ignores LV relaxation (i.e., lusitropy), which can also be deranged in sepsis [40,41]. With impaired LV lusitropy, the shape of the diastolic pressure–volume relationship is such that incremental volume significantly augments filling pressure [42]. In these patients, preload risks pulmonary edema, with little SV benefit [43].

While basic CCE offers rapid, morphological evaluations suitable for narrowing the differential diagnosis of circulatory failure, using basic CCE for specific therapeutic direction (e.g., IV fluids) has caveats. To better weigh the risks and benefits of IV fluids, Doppler ultrasound may be deployed.

## 3. Doppler Ultrasound

An eponym for the Austrian physicist—Christian Doppler—who first described the effect, Doppler ultrasound is ordinarily added to conventional, gray-scale, brightness mode (i.e., B-mode) ultrasound [44]. B-mode is the familiar, imaged-forming ultrasound that establishes basic CCE, described above. Doppler ultrasound, on the other hand, takes two general forms—color and spectral. Either of these may be added to B-mode to generate duplex or triplex ultrasound, depending on the number of components in the final examination. Color Doppler affords qualitative information on the presence, location and direction of blood flow. By convention, blood moving away from or towards the transducer is blue or red, respectively; differences in velocity are graded by color saturations—increasingly turquoise away from and yellow towards the transducer.

**Figure 2:**
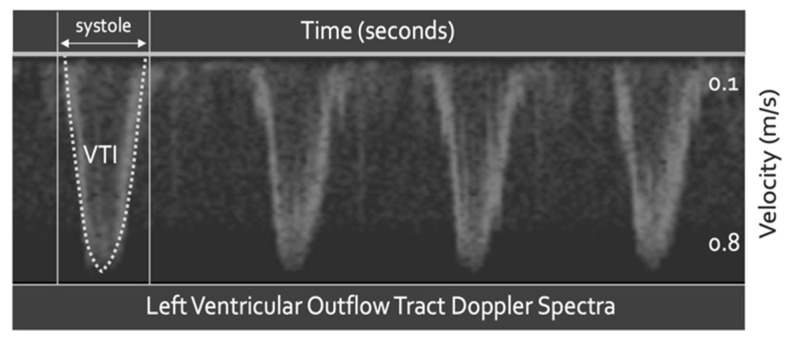
Doppler ultrasound of the left ventricular outflow tract; 4 cardiac cycles obtained via transesophageal echocardiography. The dotted trace is the maximal velocity and the area under that trace is the velocity time integral (VTI).

Spectral Doppler quantifies tissue velocity via the Doppler equation. Most commonly, the tissue of interest is blood; however, other moving tissue, such as cardiac muscle, may also be targeted [40]. Spectral Doppler measures tissue velocity in centimeters per second (cm/s) versus time (Figure 2). The area under this velocity–time curve (i.e., the velocity time integral) is distance, in centimeters (cm). If the distance travelled by blood (i.e., in cm) is multiplied by the cross-sectional area of the vessel in which the blood moves (i.e., in cm^2^), the result is volume (i.e., cm^3^); at the left ventricular outflow tract (LVOT), this calculation confers SV(Figure 2) [45,46]. Quantifiable change in SV is a powerful tool when making therapeutic decisions, especially with respect to IV fluids [47].

Though beyond the scope of this brief introduction, spectral Doppler is obtained by two fundamentally different methods—continuous wave (CW) and pulse wave (PW) [48]. As the name implies, CW Doppler continuously transmits and receives reflected ultrasound waves, making it ideal for capturing all populations of moving red blood cells within an insonation volume. Its continuity also precludes sampling ambiguity, that is, there are no velocity measurement ‘gaps’ over time. The key draw back to CW Doppler is that it lacks anatomical resolution; it cannot be directed to a specific depth within the body. Conversely, PW Doppler has excellent depth resolution; a fixed depth is localized as a function of time between the pulses. The drawbacks to PW Doppler are the converse of CW. PW does not capture the entire population of moving red cells and there is velocity sampling ambiguity (i.e., aliasing) because of the ‘gaps’ inherent to pulsing ultrasound waves. Fundamentally, this is analogous to Heisenberg’s uncertainty principle—for a moving object, there is a trade-off between certainty of location and velocity.

## 4. Advanced Critical Care Echocardiography with Spectral Doppler Ultrasound

**Figure 3:**
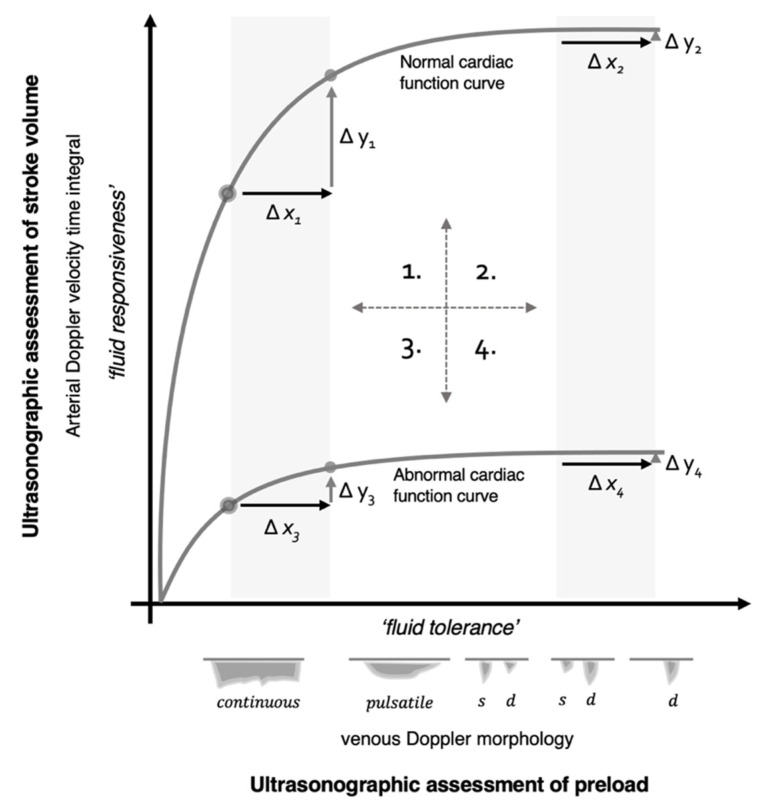
Physiological Framework linking fluid ‘tolerance’ and ‘responsiveness’. See text for details. The graph is split into quadrants highlighting 4 physiological states where changing preload (∆x, fluid tolerance) compares with changing stroke volume (∆y, fluid responsiveness ). Doppler surrogates for preload may be right-heart based (e.g., systemic vein Doppler velocimetry, VExUS) or left-heart based (e.g., pulmonary vein Doppler velocimetry, E/e’ ratio, etc.).

Advanced CCE is at the core of functional hemodynamic monitoring (FHM) [20]. FHM is founded upon measuring the slope of the cardiac function curve [49]. As considered in more detail below, measuring only cardiac filling pressure (e.g., CVP or Ppao) does not sufficiently predict how the heart will respond to additional preload [50,51,52,53]. Physiologically, this is because measuring only the *x*-axis of the Frank–Starling–Sarnoff relationship (i.e., filling pressure or volume) is agnostic to the *y*-axis (i.e., SV); accordingly, the slope (∆y/∆x) cannot be known (Figure 3). Because the clinical question being asked is how the *y*-axis (i.e., SV) will change in response to IV fluids, some measure of SV, or surrogate, must be obtained. As described above, spectral Doppler ultrasound helps achieve this goal. Accordingly, the addition of Doppler to CCE offers the clinician both functional and morphological evaluations and is, thus, classified as ‘advanced CCE’ [20].

### 4.1. Fluid Responsiveness

There are a number of mechanisms by which Doppler ultrasound illuminates the slope of the cardiac function curve (Figure 3). Fundamentally, these techniques employ arterial Doppler ultrasound, as either a direct measure—or a surrogate for—changing SV (i.e., ∆y), in response to preload variation (i.e., ∆x). With this, the advanced critical care echocardiographer surmises the slope (∆y/∆x). One general approach measures SV variation in response to cyclical preload changes generated by mechanical ventilation [54]. When the heart is operating on the ascending section (i.e., large ∆y/∆x) of its function curve, there is relatively great SV variation during the respiratory cycle; the heart is said to be ‘preload dependent’. Accordingly, when SV varies significantly during mechanical ventilation, the clinician is more confident that additional preload will augment SV. On the other hand, when the heart operates on its flat segment (i.e., small ∆y/∆x), there is less SV variation during the respiratory cycle and the heart is labeled ‘preload independent’. When this is observed, the likelihood that SV rises with further IV fluids is small [55]. Both respiratory variation in peak aortic Doppler velocity [56] and velocity time integral [57] predict SV response to IV fluids. Importantly, however, there are a number of caveats when using respiratory variation in SV to infer the slope of the cardiac function curve. These caveats include the following: active respiratory effort by the patient, relatively small tidal volume, significant changes in pulmonary (e.g., ARDS) or thoracic (e.g., obesity, open thorax) compliance, arrhythmia (e.g., atrial fibrillation), elevated right ventricular afterload and high heart rate to respiratory rate [50].

Because SV variation elicited by the respiratory cycle is sensitive to single-beat irregularities, as well as non-standardized pleural pressure change, uniform, provocative maneuvers that span multiple cardiac cycles address some of these limitations. For instance, ventilator occlusion tests modify preload via a 15-s hold at end-expiration or with an additional hold at end-inspiration [58,59,60]. These maneuvers increase and decrease preload, respectively, which when combined with the Doppler assessment of SV, test the slope of the cardiac function curve. Finally, preload can be increased via a mini fluid challenge or passive leg raise with simultaneous Doppler SV assessment [61,62,63,64,65], informing the clinician about the heart’s ability to respond to IV fluids.

In its purest sense, Doppler evaluation of the cardiac function curve necessitates calculation of the SV change (i.e., ∆y), which can be done using the LVOT velocity time integral [63,65], though distal arteries have been studied as SV surrogates, including the descending aorta [64], carotid [66,67,68] and femoral arteries [50]. Regardless of which artery is interrogated, each of the techniques described above quantify only the *y*-axis of the Frank–Starling–Sarnoff relationship, while the *x*-axis (i.e., changing preload, ∆x) is assumed and unmeasured. Arguably, however, emphasizing only ∆y favors resuscitation strategies where IV fluid is held, only when SV ceases to increase, instead of when preload (∆x) intensifies. Consequently, IV fluid provision, agnostic to cardiac filling, imperils venous congestion. Therefore, there is renewed interest in gauging preload with Doppler ultrasound, such that the hemodynamic risk of additional IV fluid is appraised independently of SV behavior. This emerging application of advanced CCE might be categorized under the more general term ‘fluid tolerance’, as described next.

### 4.2. Fluid Tolerance

Though not specified above, fluid *responsiveness* is defined by an increase in stroke volume (or cardiac output) of 10–15%, with the provision of IV fluids [69]. Whereas fluid responsiveness has a consistent, quantitative definition, fluid *tolerance* is more qualitative and unformulated [47]. The concept of fluid tolerance has arisen in step with whole-body ultrasonography and the increasingly recognized stigmata of excessive IV fluid when scanning different organ systems [70]. Though a universal definition is yet to be articulated, for the discussion herein, fluid tolerance is defined as *the capacity to accept additional IV fluids without adverse reaction*. While the extent of ultrasonographic fluid tolerance is an organ-specific concept (e.g., diffuse b-lines in the lung), to narrow the focus of the framework explicated below, only hemodynamic facets of fluid tolerance (with emphasis on venous Doppler ultrasonography) are considered further.

**Figure 4:**
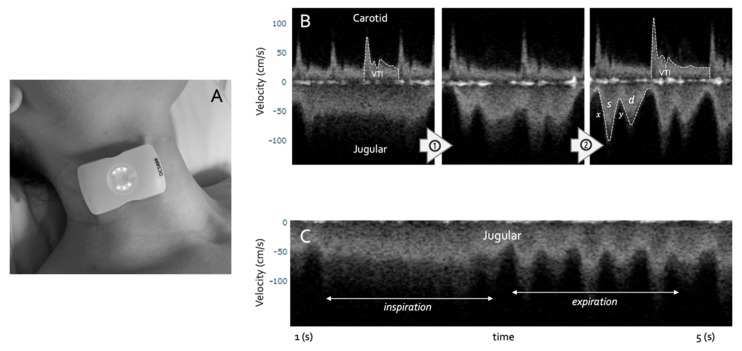
Simultaneous venous and arterial Doppler from a wearable ultrasound. (**A**) shows the wearable technology on a healthy volunteer. (**B**) shows the effect of rising preload from left to right (arrows 1 & 2 are ↑ preload). The jugular wave is amorphous indicating jugular collapse, as preload rises, the morphology of the jugular vein becomes more pulsatile and forms the s and d waves described above; the *x* and *y* refer to the pressure descents of the CVP. The carotid VTI also rises from left to right suggesting that the SV is rising with preload (e.g., quadrant 1 to 2, Figure 3). (**C**) shows just the jugular morphology from inspiration to expiration, intimating jugular collapse on inspiration.

Doppler ultrasonography of a large vein is qualitatively and quantitatively distinct from that of a large artery. When a vein is collapsed, because its intraluminal pressure is low and/or its extraluminal pressure is high, venous Doppler demonstrates a relatively indistinct, high-velocity pattern that varies with respiration; this is discussed and illustrated in subsequent sections (see Figure 4). As the pressure within the vein rises and the vein rounds out, Doppler velocity falls and takes on a pulsatile pattern, which is an inverse representation of the CVP waveform [71,72]. At end-diastole, venous Doppler velocity falls, coincident with atrial kick (i.e., the ‘a wave’). With the x-descent (i.e., falling CVP from tricuspid valve systolic excursion), venous Doppler velocity rises sharply, forming the systolic, ‘s wave’. Similarly, falling CVP during the y-descent (i.e., when the tricuspid valve opens) is marked by increased diastolic Doppler velocity, the ‘d wave’.

As central venous volume rises and stretches the atrium to its elastic limits, the pattern of the CVP and venous Doppler also change. For instance, as CVP rises, the *y*-descent exceeds the *x*-descent; accordingly, the venous Doppler ‘d wave’ eclipses the ‘s wave’. If right ventricular function is impaired, and especially if there is tricuspid regurgitation, then the venous Doppler ‘s wave’ can disappear completely, leaving only a monophasic ‘d wave’. This progression of venous Doppler, with rising CVP and/or worsening right ventricular function, has been observed in the jugular vein [73], superior and inferior vena cavae [74,75,76], hepatic vein [77], femoral vein [78] and even within intra-renal veins [79]. Indeed, Iida and colleagues recently noted a direct relationship between CVP and the qualitative intra-renal venous Doppler morphology described above (see *x*-axis of Figure 3 and Figure 4B) [79,80].

To standardize some of the aforementioned physiology, the venous excess ultrasound score (VExUS) was proposed [81]. The VexUS score combines Doppler ultrasonography of the hepatic and portal veins and the size of the IVC into a composite grade between zero and three. Higher VexUS is associated with kidney injury, which mirrors earlier data linking high CVP to renal dysfunction in congestive heart failure [82,83,84]. Though not included in VexUS, the pulmonary venous Doppler waveform transforms analogously to systemic veins with left atrial pressure elevation [85]. Further, the ratio of early trans-mitral filling velocity (i.e., the E wave) to spectral tissue Doppler (i.e., e’) rises with left atrial pressure [86]. Accordingly, one can imagine a scoring system akin to VexUS for the left heart, such that pulmonary venous congestion is standardized and stratified.

The ultrasonographic findings of venous congestion speak to an aspect of fluid tolerance, given the definition offered above. That is, venous congestion intimates diminished capacity to accept additional IV fluid and could be an adverse hemodynamic pattern ascribed to excessive fluid resuscitation. Nevertheless, while venous congestion is remarkable when present, its absence does not necessarily affirm that further IV fluid will be tolerated. Fundamentally, this is because the venous ultrasonography detailed above informs only the ∆x (i.e., preload) of the cardiac function curve. As such, a clinician who singly follows hemodynamic measures of fluid tolerance (∆x) ignores how the heart responds to IV fluids. Conversely, a clinician who assesses only how the heart responds to IV fluids (∆y) misses warnings of preload excess. In the framework described below, it is maintained that the advanced sonographer pays equal notice to both axes of the cardiac function curve.

## 5. Physiological Framework

The foundations of the framework to be discussed below have already been laid out above. During advanced CCE, the sonographer need not subscribe to monitoring either ‘fluid responsiveness’ or ‘fluid tolerance’; rather, the sonographer deploys Doppler ultrasound to assess both when deliberating IV fluid administration. The reasons are illustrated by examining two hypothetical cardiac function curves, represented in Figure 3. These two curves embody physiological extremes; they might depict different patients or a single patient within the course of critical illness. Furthermore, there are an infinite number of intermediary curves existing between those illustrated, necessarily over-simplifying this discussion. Nevertheless, the analysis illuminates the physiological and clinical links between sonographic measures of fluid ‘tolerance’ (i.e., preload, *x*-axis) and ‘responsiveness’ (i.e., SV, *y*-axis), using the Frank–Starling–Sarnoff relationship as a unifying principle.

### 5.1. Quadrant 1

To break down the examination, Figure 3 is parsed into quadrants. The first quadrant (upper left) is considered ‘normal’ physiology; there is a steep relationship between changing preload and SV. A patient in this position is referred to as operating on the ‘ascending’ or ‘preload dependent’ portion of the Frank–Starling curve, because raising preload (∆x) with IV fluids significantly increases SV (∆y). In addition, measures of venous Doppler herald low preload in this state; therefore, fluid ‘responsiveness’ and ‘tolerance’ are concordant.

### 5.2. Quadrant 2

The second quadrant (upper right) discloses a patient with normal underlying cardiac function who is, nonetheless, operating on the ‘flat’ or ‘preload independent’ portion of the Frank–Starling curve. Ostensibly, this occurs in a patient with a normal heart, who is ‘over-resuscitated’ by IV fluids. Arriving at this state is a criticism leveled against protocols that administer preload until fluid responsiveness disappears; there is data to support this concern [87]. For example, some algorithms for goal-directed therapy during elective surgery ‘optimize’ preload by encouraging IV fluid until fluid responsiveness is extinguished. Following this approach, excessive fluid and length-of-stay were observed in patients with the greatest cardiovascular reserve, ostensibly those with the most upright cardiac function curves [88]. Therefore, impelling patients until (∆y) vanishes without regard to climbing preload (∆x), may be deleterious. Finally, in this quadrant, because giving preload does not significantly increase SV and venous Doppler exhibits elevated preload, fluid ‘unresponsiveness’ and ‘intolerance’ are conceptually concordant.

### 5.3. Quadrant 3

With diminished cardiac function, the third quadrant (lower left) demonstrates discordance between ultrasonographic signs of fluid tolerance and responsiveness. That is, the patient appears fluid tolerant via venous Doppler, despite being fluid unresponsive by arterial Doppler. Arguably, this patient meets the definition of fluid intolerance—a diminished capacity to accept additional IV fluid without adverse reaction—but this is a state of *dynamic fluid intolerance,* because it is expressed only by performing a dynamic assessment of SV (∆y). A clinical example of this quadrant is a patient with septic cardiac dysfunction and concurrent sepsis-associated venodilation (± volume depletion), engendering low preload; this pathophysiological pattern is well-reported. Magder and Bafaqeeh observed that 25% of critically ill patients with a CVP of 0–5 mmHg were fluid unresponsive [89], while a meta-analysis noted that 40% with a CVP less than 8 mmHg were fluid unresponsive [34]. Despite conflicting evidence for size and collapsibility of the IVC, one of the more favorable investigations found that roughly 20% of patients with IVC collapse were fluid unresponsive [90]. Consequently, dynamic fluid intolerance is likely common.

### 5.4. Quadrant 4

In the fourth quadrant (lower right), there is conceptual concordance between fluid intolerance and unresponsiveness. An example of this state might be the septic patient described above, who began in quadrant 3 with low preload and then received IV fluids. Physiologically, this would only raise filling pressure if there was no concomitant increase in cardiac function. It must be reiterated, however, that this physiological framework is over-simplified, assuming that the slope of the cardiac function curve does not change, consequent to additional preload [91,92].

## 6. Discussion

Given the framework above, the foremost implication for current practice is that certainty around the slope of the cardiac function curve stipulates a dynamic assessment. While it is tempting to use LVEF as a surrogate for cardiac function, this too falls short; the LVEF can appear normal when cardiac function is reduced. This assertion is illustrated by an investigation by Mahjoub and colleagues, where 83, septic, critically ill patients were studied for fluid responsiveness with concomitant echocardiographic parameters of diastolic function [93]. At baseline, fluid responders and non-responders had no significant difference in LVEF (i.e., mean > 50% in both groups). Further, there was no significant difference in sonographic measures of left heart filling pressure or diastolic function at baseline. Notably, however, the non-responders significantly increased the ratio of early diastolic filling to tissue Doppler (i.e., E/e’ ratio, a marker of left atrial pressure), while the fluid responders did not increase this ratio. While this obviously reflects the left heart, it nonetheless reiterates the physiology in Figure 3. The fluid non-responders had normal LVEF at baseline but exhibited significant expansion along the *x*-axis following IV fluids (e.g., moving from quadrant 3 to 4). By contrast, the fluid responders did not significantly increase Doppler measures of preload with IV fluids. A separate examination by Du and colleagues evaluated hepatic venous wave morphology between volume responders and non-responders [94]. Again, in line with the physiological framework summarized in Figure 3, only fluid non-responders significantly augmented hepatic vein d-wave velocity following IV fluid expansion; this is anticipated with rising right atrial pressure (e.g., quadrant 3 to 4).

Another implication of the framework presented above is that it explains the variable accuracy of preload surrogates to detect fluid responsiveness [36,90,95,96]. Note that the shape of the cardiac function curve recapitulates the receiver operator curve for using preload (e.g., IVC variation, VexUS) to predict SV response (i.e., fluid responsiveness). If a study population is entirely comprised of patients with normal cardiac function, then low preload will accurately predict volume responsiveness (i.e., quadrant 1, sensitivity) and high preload will accurately predict volume unresponsiveness (i.e., quadrant 2, specificity). Yet, by first principles, finding a strong relationship between preload and preload responsiveness only demonstrates an investigation with inadequate patient heterogeneity. Accordingly, to the extent that a study includes patients with increasingly flat cardiac function curves, the relationship between preload and preload responsiveness becomes less definite; this is analogous to a flattening receiver operator curve. There will be a greater fraction of ‘false positives’ (e.g., quadrant 3) and false negatives when a study includes a broad spectrum of cardiac function.

**Figure 5:**
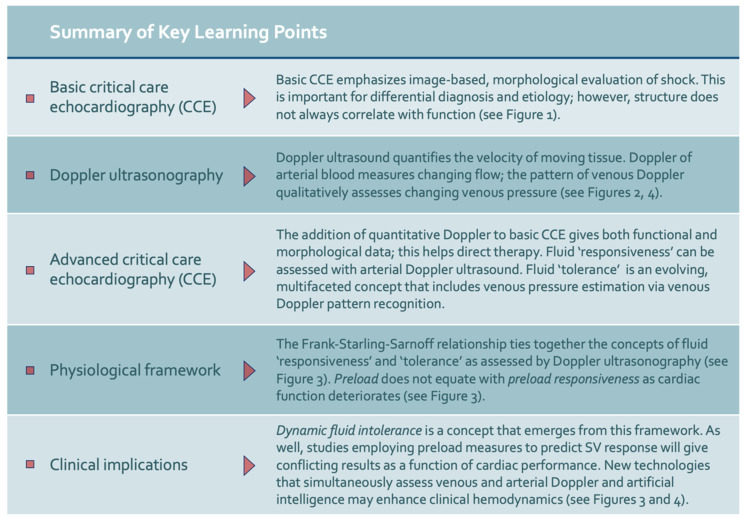
Summary of key learning points.

A final implication of this novel framework is that emerging technology may help automate ultrasonographic assessments of both preload (e.g., venous Doppler) and stroke volume (e.g., arterial Doppler), *simultaneously* (Figure 4) [97]. We have developed a wireless, wearable Doppler ultrasound that concurrently insonates the jugular vein and common carotid artery [98,99,100,101,102,103,104]. To our knowledge, we have first reported synchronous venous and arterial Doppler during a dynamic assessment, both in a volume-responsive, healthy volunteer and critically ill, septic patient [99]. The healthy volunteer demonstrated venous and arterial Doppler changes consistent with quadrant 1 physiology, while the critically ill patient manifested quadrant 3 physiology—dynamic fluid intolerance. Theoretically, a large dataset of synchronously acquired venous and arterial Doppler measurements could be used for machine learning-powered ‘clustering’ akin to a recent, provocative investigation by Geri and colleagues that elucidated unique, ultrasonographic septic ‘phenotypes’ [15]. Figure 5 summarizes these implications as well as other key learning points from this review.

## 7. Conclusions

Ultrasound has become the dominant hemodynamic assessment tool within the emergency department and intensive care unit. While basic, morphological studies help diagnose and triage, they are less precise regarding therapy, especially IV fluids. With the addition of Doppler ultrasonography to gauge cardiac filling and output, the clinician adopts an advanced approach, appraising fluid ‘tolerance’ and ‘responsiveness’, respectively. The physiological framework described above is founded by the Frank–Starling–Sarnoff curve, conceptually linking fluid ‘tolerance’ and ‘responsiveness’. This model emphasizes the dynamic nature of the cardiac function curve; with worsened performance, the relationship between preload and SV response is less clear cut. Because the ejection fraction does not definitively judge preload dependence, anticipating the response to additional IV fluid requires a dynamic measure. Emerging ultrasound technology may facilitate these decisions and open new and exciting avenues of research, especially in conjunction with large datasets and machine learning.

## Data Availability

Not applicable.
